# Crayfish population size under different routes of pathogen transmission

**DOI:** 10.1002/ece3.9647

**Published:** 2023-01-06

**Authors:** Mikko Koivu‐Jolma, Raine Kortet, Anssi Vainikka, Veijo Kaitala

**Affiliations:** ^1^ Department of Physics, Faculty of Science University of Helsinki Helsinki Finland; ^2^ Department of Environmental and Biological Sciences University of Eastern Finland Joensuu Finland; ^3^ Organismal and Evolutionary Biology Research Programme, Faculty of Biological and Environmental Sciences University of Helsinki Helsinki Finland

**Keywords:** *Aphanomyces astaci*, cannibalism, contact infection, crayfish plague, density‐dependent transmission, environmental infection, force of infection, frequency‐dependent transmission, incidence rate, population dynamics model, population regulation, scavenging

## Abstract

We present an epidemiological model for the crayfish plague, a disease caused by an invasive oomycete *Aphanomyces astaci*, and its general susceptible freshwater crayfish host. The pathogen shows high virulence with resulting high mortality rates in freshwater crayfishes native to Europe, Asia, Australia, and South America. The crayfish plague occurrence shows complicated dynamics due to the several types of possible infection routes, which include cannibalism and necrophagy. We explore this complexity by addressing the roles of host cannibalism and the multiple routes of transmission through (1) environment, (2) contact, (3) cannibalism, and (4) scavenging of infected carcasses. We describe a compartment model having six classes of crayfish and a pool of crayfish plague spores from a single nonevolving strain. We show that environmental transmission is the decisive factor in the development of epidemics. Compared with a pathogen‐free crayfish population, the presence of the pathogen with a low environmental transmission rate, regardless of the contact transmission rate, decreases the crayfish population size with a low risk of extinction. Conversely, a high transmission rate could drive both the crayfish and pathogen populations to extinction. High contact transmission rate with a low but nonzero environmental transmission rate can have mixed outcomes from extinction to large healthy population, depending on the initial values. Scavenging and cannibalism have a relevant role only when the environmental transmission rate is low, but scavenging can destabilize the system by transmitting the pathogen from a dead to a susceptible host. To the contrary, cannibalism stabilizes the dynamics by decreasing the proportion of infected population. Our model provides a simple tool for further analysis of complex host parasite dynamics and for the general understanding of crayfish disease dynamics in the wild.

## Graphical abstract

Many European crayfish populations have been decimated by the invasive crayfish plague *Aphanomyces astaci*, already harbored to Europe, Asia, and Australia. To contribute to the discussions of the means to control the disease, a population dynamical model is presented to build the theory to master the crayfish population dynamics and the disease dynamics.

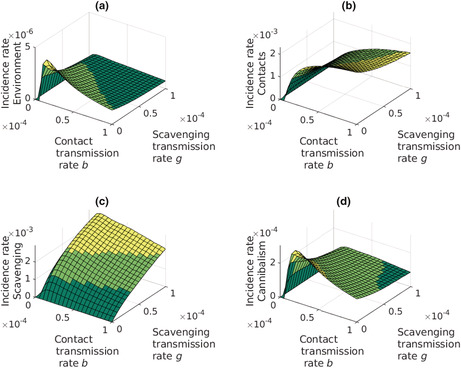

## 1 INTRODUCTION

Pathogens and their hosts are tied together. Given enough time, those pairs that can coexist thrive, while the others disappear through extinction of either the host or the pathogen. An introduction of a novel pathogen to an established ecosystem may bring forth rapid changes in the population dynamics of the naïve hosts. If the pathogen can spread efficiently even within a sparse host population, for example through water, the effects are even stronger. Such an invasion of a pathogen may be disastrous for most host populations, as has been in the case of the oomycete *Aphanomyces astaci*, causing deadly crayfish plague in the European noble crayfish (*Astacus astacus*; Makkonen et al., 2018; Martín‐Torrijos et al., 2021; Svoboda et al., 2017).

While crayfishes are generalists with occasionally high biomass (Reynolds et al., 2013), their population dynamics are complex and affected by a multitude of mechanisms. Crayfishes, often considered keystone species, show ontogenetic and seasonal niche shifts (Abrahamsson, 1966; Guan & Wiles, 1998) and act as ecosystem engineers by digging burrows and clearing aquatic vegetation (Abrahamsson, 1966; Dorn & Mittelbach, 1999; Statzner et al., 2000; Thomas & Taylor, 2013). Importantly, crayfish scavenge a wide range of dead animals including conspecifics and other crayfishes (Guan & Wiles, 1998; Houghton et al., 2017). Scavenging can increase per capita resource availability by widening the potential scope of food items (Boros et al., 2020), but crayfish also commonly show cannibalism at high population densities (Guan & Wiles, 1998; He et al., 2021; Houghton et al., 2017). Cannibalism can efficiently regulate population growth and induce fluctuating dynamics both in the average size of individuals and in the population size in indeterminately growing organisms (Abrahamsson, 1966; Claessen et al., 2004; Houghton et al., 2017; Rudolf & Antonovics, 2007). The relative abundance of suitable refugees affects the susceptibility of crayfish to cannibalism at all ages (Guan & Wiles, 1998; Houghton et al., 2017). Crayfish are predated not only by larger conspecifics but also by numerous fishes, mammals and birds (Houghton et al., 2017; Reynolds et al., 2013), and large crayfish are harvested as part of freshwater fisheries or “crayfisheries”. All these mechanisms challenge the development of simple population models for crayfishes, while such models would be needed for the sustainable management of these species (Jones & Coulson, 2006; Todd et al., 2018; Whiterod et al., 2020).

A major complication to the modeling of crayfish population dynamics arises from their diseases that can cause both chronic sublethal fitness losses and acute, catastrophic population crashes. Individuals weakened by diseases may be increasingly vulnerable to cannibalism, while dead individuals may be readily consumed. Both feeding habits can act as direct transmission routes for the pathogens and further complicate the epidemiological and population dynamical patterns in crayfish and their pathogen populations (e.g., Getz & Pickering, 1983; Kohler & Holland, 2001; Rudolf & Antonovics, 2007). Of all the known crayfish disease agents, oomycete *A. astaci* can be considered the most virulent (Makkonen et al., 2018; Martín‐Torrijos et al., 2021; Svoboda et al., 2017). *A. astaci* originates from North America, where it coexists with its native host species such as signal crayfish *Pacifastacus leniusculus*. The crayfish plague manifests in physiological and behavioral changes, progressive paralyzation, and ultimately death, but the disease severity may vary significantly among host species with native host species being able to live even several years with the pathogen (Jussila et al., 2021; Oidtmann et al., 2002). Crayfish infected with *A. astaci* spread the pathogen by carrying the oomycete mycelium in their cuticle. The mycelium releases the infective zoospores to the water (Oidtmann et al., 2002). The number of zoospores that are released depends on the status of the infected crayfish. If the infection is latent, i.e., weak (asymptomatic), and when the crayfish has control over the pathogen, zoospore production remains low but may increase during molting (Strand et al., 2012). However, for a period that begins 1 week before the disease‐induced death and continues some days after the death, the host zoospore production increases. Zoospores are initially distributed in water unequally so that the density is the highest close to the source host. Without significant turbulence, zoospores tend to remain near the bottom of the waterbody but use chemotaxis to find the crayfish (Oidtmann et al., 2002; Strand et al., 2012). Carcasses of succumbed crayfish can remain infective several days after the death and release new zoospores into the environment. Active zoospores have limited survival time outside host, but they can transform into resistant cysts that in turn can transform back to motile zoospores (Oidtmann et al., 2002; Unestam, 1969). The cysts have considerably longer lifespans than the active zoospores, surviving 2 weeks in distilled water (Unestam, 1969). Cold environment increases the survival of the spores, and a survival period of 2 months has been observed (Unestam, 1966).

Although there has been a great empirical interest in the epidemiology of crayfish plague and population dynamics of crayfish stocks, these dynamics have not been captured by developing host parasite models that would explicitly account for the various infection routes (Jussila et al., 2015; Strand et al., 2012). To formulate such a model, it is necessary to describe the transmission dynamics of the *A. astaci*. First, the spread of the plague in a naïve crayfish population may largely follow standard density‐dependent transmission with the resulting fast population crash. However, under a certain threshold density, the pathogen transmission might follow a horizontal or vertical, frequency‐dependent infection process regulated by the behavior of the crayfish (Svoboda et al., 2017). Thus, transmission of the crayfish plague generally includes an environmental transmission component through the environmentally spreading spores emitted by the dead and alive infected crayfish (Oidtmann et al., 2002) and contact‐aided transmissions with varying relative importance. Second, contacts conveying transmission may relate to aggressive and territorial behavior of crayfishes and to the scavenging of the deceased carcasses, a portion of which are infected with *A. astaci*. Cannibalism has been shown to be an important transmission route, for example, for the Yellow head virus in shrimp aquaculture (Hamano et al., 2015), but it is unclear, if cannibalism is a significant infection route in wild populations (Rudolf & Antonovics, 2007). The third impetus for the pathogen transmission comes through scavenging. Crayfish have been shown to eat infected pieces of conspecifics (Imhoff et al., 2012; Oidtmann et al., 2002). Therefore, the crayfish do not avoid the infected carcasses, but use them as a food source. The necrophagy increases the risk of transmission by two mechanisms. First, the crayfish are in close contact with the infected carcasses for a prolonged time. Because *A. astaci* zoospore production is at highest right before and after the death of the infected individual, the effect of scavenging in pathogen transmission is likely notable for the scavenging individual. Second, transmission through eating infected parts of conspecifics can result in higher pathogen concentration than transmission through environment, as shown for the microsporidian parasite infecting two freshwater host species by Imhoff et al. (2012). As a result, the most obvious transmission route for *A. astaci* remains the environment, but the possibility of significant contact transmissions cannot be ruled out.

The abovementioned infection routes are hereafter referred to as environmental transmission (Keeling & Rohani, 2002; Li et al., 2009), contact transmission (Keeling & Rohani, 2002), transmission through cannibalism (Rudolf & Antonovics, 2007), and transmission through scavenging (Oidtmann et al., 2002), according to the source of infection. The environmental transmission is considered as a density‐dependent process, whereas the contact transmission, transmission through cannibalism, and transmission through scavenging are modeled as density and frequency‐dependent processes. The assumption behind this is the idea that the crayfish cannot tell apart the health status of their live or deceased conspecifics. A certain fraction of the cannibalized or otherwise contacted individuals and scavenged dead individuals are infectious leading to infection of a susceptible individual.

Because the zoospore density in the proximity of the susceptible hosts is the determinant biological route to infection, equal exposure may be obtained through various transmission routes, and the relative role of each transmission route may depend on host population density. For the disease transmission, on the other hand, the disease virulence is the most important trait as predicted by the classic trade‐off model of disease virulence (Anderson & May, 1982). However, due to the scavenging, high infection mortality does not prevent the infection from spreading. Therefore, the assumptions of the trade‐off model do not hold (Anderson & May, 1982), and transmission is expected even to rare hosts via scavenging. These mechanisms are likely to cause highly labile population dynamics for which there is no standard population dynamical theory (Alizon et al., 2009). The present model is applicable to species that show relatively stable mortality rate throughout the life and across seasons (including *A. astaci* and similar species). For example, *A. astacus* has a lifespan of over 15 years, though the estimates vary greatly (Vogt, 2014). It generally grows slower and lives longer in higher latitudes and colder environments.

We focus here on the population dynamical consequences of the four transmission routes using a model with two host life stages that can both be susceptible or infected. We analyze the effects of different transmission routes on the long‐term crayfish population density, which is measured as the corresponding mean value of the variable under consideration (because sometimes the system is cyclic, see Materials and Methods). We compare the environmental transmission to other transmission routes to uncover if some routes dominate the others and if they have a joint effect on the crayfish population. Moreover, we examine specifically if the transmissions through scavenging or cannibalism have an effect of the crayfish population size. We quantify the intensity of infection in the terms of incidence rate of the plague infections in the adult crayfish population. In this measure, the population sizes represent the susceptible and the infected adult crayfish populations. Our goal is to provide a generally applicable model of a fatal disease to assess the relative importance of different transmission routes in any structurally similar system with cannibalistic hosts.

## 2 MATERIALS AND METHODS

### 2.1 Model description

We consider here a simplified crayfish population with two life stages (juveniles and adults). Two behavioral patterns are characteristic for the crayfish in their acquisition of resources: (1) intraspecific predation (cannibalism) and (2) scavenging. The crayfish population is subject to a disease that has four transmission routes: (1) environmental transmission, (2) contact transmission, (3) transmission through cannibalism, and (4) transmission through scavenging carcasses of disease‐killed individuals.

**FIGURE 1:**
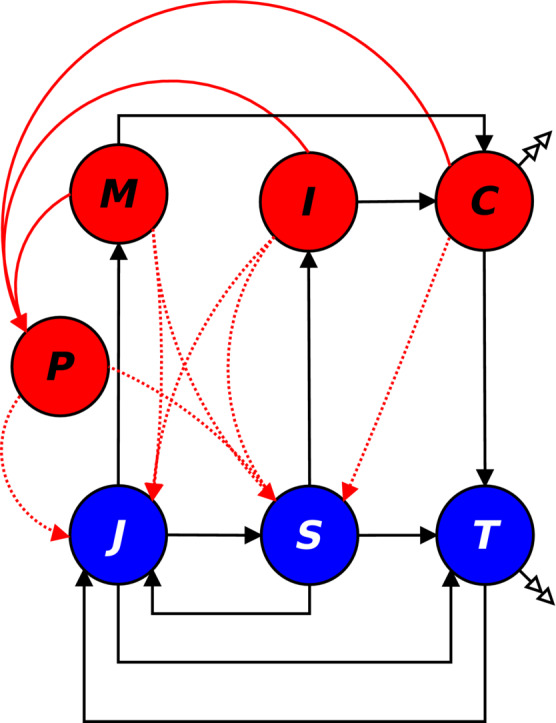
The crayfish and *Aphanomyces astaci* form a complex system with several connections. The crayfish population is regulated by the intraspecific predation, where susceptible adult crayfish (*S*) cannibalize the juveniles (*J*). Susceptible adults also consume the crayfish carcasses (*T*) for additional energy. Part of the energy thus gained can be used in reproduction. The oomycete grows in the infected juveniles (*M*), adults (*I*) and carcasses (*C*) producing infective spores (*P*). The susceptible juveniles are infected either through the water or through contact with infected conspecifics. Likewise, the susceptible adults can get an infection through water and through contact with infected conspecifics. Additionally, adults are at risk of getting infected through intraspecific predation and necrophagy. Infected carcasses lose their infectiveness at certain rate, transferring from (*C*) to (*T*). Both types of carcasses decay naturally. The blue circles signify the pathogen‐free compartments in the model, whereas the red circles mark the pathogen affected compartments. Solid black lines show the direct development or reproduction of the individuals, and dotted red lines represent the infection transmission routes.

The density of the plague spores in the environment is denoted by *P* (Figure 1). The densities of susceptible juvenile and adult crayfish are denoted by *J* and *S*, respectively. *M* and *I* denote the densities of infected juvenile and adult crayfish. *T* and *C* denote the densities of the deceased uninfected and infected crayfish carcasses, respectively (Figure 1).

The general model of the crayfish population dynamics, infected by the crayfish plague, is described by a set of differential equations as

(1) $$\frac{\mathrm{dP}}{\mathrm{dt}}=\mathrm{aM}+\mathrm{aI}+\mathrm{dC}-\gamma_{P}P$$

(2) $$\frac{\mathrm{dJ}}{\mathrm{dt}}=r_{S}S-\mu_{J}J-r_{J}J-\mathrm{cSJ}+\text{wcSJ}+\text{wgST}-\mathrm{\alpha PJ}-\mathrm{bJI}-\mathrm{bMJ}$$

(3) $$\frac{\mathrm{dS}}{\mathrm{dt}}=r_{J}J-\mu_{S}S-\mathrm{\alpha PS}-\mathrm{gSC}-\mathrm{cSM}-\mathrm{bSI}-\mathrm{bSM}$$

(4) $$\frac{\mathrm{dM}}{\mathrm{dt}}=\mathrm{\alpha PJ}+\mathrm{bIJ}+\mathrm{bMJ}-\mathrm{cSM}-\mu_{J}M-\mu_{\inf}M$$

(5) $$\frac{\mathrm{dI}}{\mathrm{dt}}=\mathrm{\alpha PS}+\mathrm{gSC}+\mathrm{cSM}+\mathrm{bSI}+\mathrm{bSM}-\mu_{S}I-\mu_{\inf}I$$

(6) $$\frac{\mathrm{dT}}{\mathrm{dt}}=\mu_{J}J+\mu_{S}S-\mathrm{gST}-\gamma_{T}T+\gamma_{C}C$$

(7) $$\frac{\mathrm{dC}}{\mathrm{dt}}=\mu_{\inf}I+\mu_{\inf}M-\mathrm{gSC}+\mu_{S}I+\mu_{J}M-\gamma_{T}C-\gamma_{C}C$$

Infection transmissions through contacts, intraspecific predation, and necrophagy are modeled as frequency‐dependent processes and those through the environment as a density dependent process. The adjustments of the model parameters for the noble crayfish (*A. astacus*) are presented below in section “Parameterization of the model”.

The plague spores are released into the environment by the alive (*M*, *I*) and deceased (*C*) infected crayfish at rates *a* and *d*, respectively (Equation 1). The spores decay in the environment at a constant rate $\gamma_{P}$. Healthy juvenile crayfish, J, is produced by the healthy adult crayfish, *S*, at a constant rate $r_{S}$, which is the population growth rate (Malthusian parameter) of the crayfish population (Equation 2). The reproduction of the new juveniles is supported by the fraction w of the energy gained from cannibalism (cSJ) and scavenging of healthy carcasses (gST). The healthy juvenile population, J, decreases through natural mortality ($\mu_{J}$), maturation ($r_{J}$), environmental infection (αPJ), and contact transmissions $\left(\mathrm{bJI},\mathrm{bJM}\right)$. The healthy adult crayfish population, *S*, increases through the maturation of the young healthy crayfish and decreases through natural mortality ($\mu_{S}$), or getting an infection from environment (αPS), scavenging of infected carcasses (gSC), cannibalizing infected juveniles (cSM), or through the contacts with infected individuals (bSI,bSM; Equation 3). The population of infected juveniles, *M* (Equation 4), increases due to environmental and contact transmissions of the healthy juveniles (Equation 2) and decrease due to cannibalism, natural mortality and virulence, where virulence parameter $\mu_{\inf}$ describes the mortality rate due to the plague infection. The infection mortality is assumed to exceed the background mortality of both adult and juvenile crayfish. The population of infected juveniles, *I* (Equation 5), increase due to environmental transmissions, scavenging and cannibalizing infected individuals and through contact transmissions of the healthy juveniles. It decreases due to natural mortality and virulence. The pool of healthy carcasses increases through the natural mortality of the healthy individuals and decreases from being scavenged, or by decaying in the nature. Infected carcasses, *C*, lose their infectiveness at rate $\gamma_{C}$, thus turning into healthy carcasses. Infected carcasses, *C*, increase with the virulence and natural mortality of the infected individuals, and decrease from being scavenged, or through decay $\left(\gamma_{T}\right)$. The decay of the carcasses is due to the natural decomposing process as well as a result of the scavenging by the other scavenging species. Parameter g has a dual meaning: it denotes the scavenging rate of healthy and infected carcasses by the adult crayfish, and the rate of transmission from scavenging an infected carcass

The model includes several assumptions that may be important for some crayfishes or specific circumstances. In the spatial context, our model describes a closed population leaving out spatial structures and migrations. In the disease dynamics, we exclude fish and other potential vectors of transmission. We also assume that the disease has a constant virulence, i.e., represents a single non‐evolving strain. In particular, we assume that the disease is fatal such that no recovery or immunization takes place. Globally, this leaves out a few North American crayfishes as hosts, but includes the rest. We assume that there is no explicit cannibalism within the young age class. We also assume that the young class individuals rely on environmental sources of food instead of scavenging conspecifics. The only way in which the population recruits is through the linear offspring production (Equation 2), and the only way the individuals are vanished from the system is through the decay of the uninfected (Equation 6) and infected carcasses (Equation 7).

When comparing the force of infection of the different transmission routes, we use the measure of incidence rate defined as follows: Incidence rate measures the rate of appearance of new cases in the group or population, i.e., the number of infected within a specified period of time related to the total number of individuals exposed to risk during that period (Olweus, 1989). We apply this measure using the adult crayfish as the target group such that the mean rates of infections of the adult crayfish population are calculated during the last 60,000 days of the simulation. Thus, the incidence rate of each transmission route becomes:

$$\text{Environmental transmission}:\text{mean}\;\left(\mathrm{\alpha PS}\right)/\text{mean}\;\left(S\right).$$

$$\text{Contact transmission}:\text{mean}\;\left(\mathrm{bSI}+\mathrm{bSM}\right)/\text{mean}\;\left(S\right).$$

$$\text{Transmission through scavenging}:\text{mean}\;\left(\mathrm{gCS}\right)/\text{mean}\;\left(S\right).$$

$$\text{Transmission through cannibalism}:\text{mean}\;\left(\mathrm{cMS}\right)/\text{mean}\;\left(S\right).$$

### 2.2 Parameterization of the model

We apply the model on the noble crayfish. The parameters for crayfish population density, population structure, and fecundity were adapted from Abrahamsson (1966, 1971). Noro and Buckup (2008) studied the population structure of *Parastacus defossus* in Brazil, revealing similar vital rates. The parameter values for cannibalism and scavenging of the crayfish carcasses were adapted from Guan and Wiles (1998), Houghton et al. (2017), and He et al. (2021). The susceptible crayfish life spans were adapted from Vogt (2014). The effects of *A. astaci* on *A. astacus* were adapted from Oidtmann et al. (2002) and Svoboda et al. (2017) and on *P. leniusculus* from Aydin et al. (2013), Jussila et al. (2017), and Thomas et al. (2020).

Parameters for the life history of *A. astaci* were aggregated from Strand et al. (2012) and Oidtmann et al. (2002). Values for the infection rate for susceptible host were adapted from *A. astacus* data from Makkonen et al. (2014). The survival of *A. astaci* spores has not been studied in detail in natural conditions and may vary considerably (Svoboda et al., 2017). We assume that the half‐life of *A. astaci* spores is in between the estimated extremes, 14 days.

The decay of carcasses occurs in aquatic environment: carrions become edible at rate *γ*_*T*_, and the decay of the infective spores from the infected carcasses occurs at rate *γ*_*C*_. In the latter process, the infected carcasses turn into uninfective carcasses. The decay process describing the general removal of the carcasses from the environment, *γ*_*T*_, depends on the environment as it happens through the biological decomposing process and as a consequence of scavenging by other animals. Data of the decay processes in the aquatic environments are scarce. Parmenter and Lamarra (1991) observed that 80% of the total dry mass of fish was decomposed in the period of 4.5 months in a freshwater marsh environment. The carcasses were protected from scavengers, and thus, we consider *γ*_*T*_ = 0.01. The decay of the disease has been suggested to be a quicker process. Oidtmann et al. (2002) observed that *A. astaci* remains viable in a crayfish carcass for 5 days in 21°C. However, the carcasses remain infective longer in lower temperatures, and the infectivity period in natural waters has not been studied. Because infected carcasses remain usually in the cool bottom of the water body, we use a conservative estimate and use the same decay value as for the spores in the environment, *γ*_*C*_ = 0.01. This very conservative estimate assures that all spore producing mycelium has the most likely decayed from the environment. For all the model parameters, see Table 1.

**TABLE 1:** The parameters used in the numerical simulations

Parameter		Range	Unit
*r*_*S*_	Growth rate	0.01	day^−1^
*r*_*J*_	Maturation rate	0.002	day^−1^
*c*	Cannibalism rate	10^‐6^ … 2 × 10^−5^	day^−1^ individuals^−1^
*α*	Environmental infection rate	0 … 10^−9^	day^−1^ individuals^−1^
*b*	Contact infection	0 … 1 × 10^−4^	day^−1^ individuals^−1^
*g*	Infection rate from scavenging deceased carcasses	0 … 10^−4^	day^−1^ individuals^−1^
*μ*_*J*_	Juvenile mortality	0.0012	day^−1^
$\mu_{S}$	Adult mortality	0.00082	day^−1^
*μ*_Inf_	Mortality due to the infection	0.03	day^−1^
*a*	Release rate of spores from infected individuals	200	day^−1^
*d*	Release rate of spores from infected carcasses	1600	day^−1^
*γ*_*P*_	Decay of the spores	0.05	day^−1^
*γ*_*C*_	Decay of the infection in the infected carcasses	0.008	day^−1^
*γ*_*T*_	Decay of the carcasses	0.01	day^−1^
*w*	Fraction of energy gained by cannibalism and used to produce offspring	0.5	

### 2.3 Numerical methods

**FIGURE 2:**
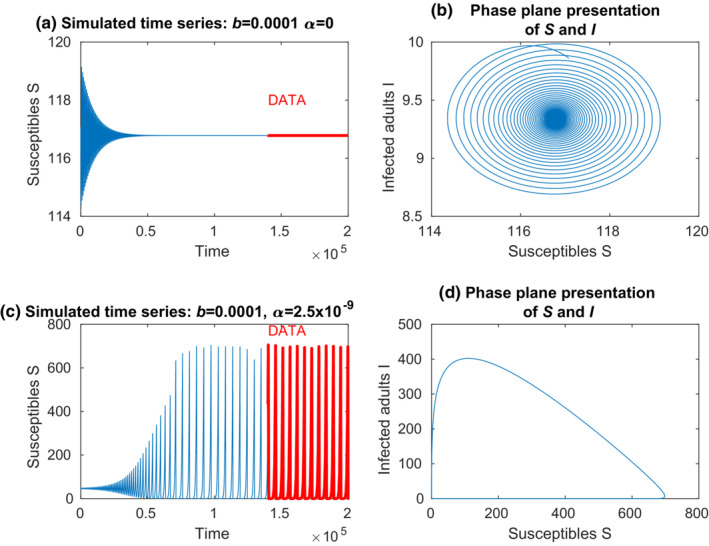
(a) an example of a locally stable population dynamics. The model was simulated over 200,000 units (days). The initial transient, the length, and the form of which may depend on the parameters and the initial values of the model (1)–(7). Thus, the initial transient is removed by accepting only the last 60,000 units of the data for the analyses (red line). These data are denoted in the analyses by *S*, *I*, etc. (b) the phase plane representation of S and I in the whole simulation data. (c) an example of a periodic population dynamics. The initial transient of the simulation outcome is removed, and the analyses are carried out using the last 60,000 units of the simulation outcome in the analyses (red line). (d) the phase plane representation of *S* and *I* in the last cycle of the data.

The numerical simulations of the model (1)–(7) were performed using the ode15s solver of Matlab R2020b and R2022a to integrate systems of stiff differential equations. The parameter ranges under investigation are presented in a discretized form on the *x*‐ and *y*‐axes of the figures. In order to obtain the data for the analyses, we simulated the model for 200,000 time units (Figure 2) and omitted the first 140,000 time units to remove the initial transients. The data, which are marked by red color, represent either locally stable dynamics (Figure 2a) or periodic oscillations (Figure 2c). The data used in the analyses are denoted as *S*, *I*, etc. The peak‐to‐peak amplitude of the data is used to tell apart locally stable time series (amplitude is zero) and periodic time series (amplitude is positive). The peak‐to‐peak amplitude is measured as the difference between the maximum and minimum values of the time series of the susceptibles, that is, max (*S*)‐min (*S*). The most of the analyses are carried out using the mean values of the data, such as mean (*S*), mean (*I*), etc.

The initial values of the simulations were determined by calculating numerically (numeric solver “vpasolve”, Matlab R 2019) the (unique) species coexistence equilibria and then adding to each equilibrium value a small disturbance. After such a small deviation from the equilibrium value stable population dynamics return to the corresponding equilibria whereas unstable population dynamics quickly develop into their characteristic fluctuations. The parameter ranges showing locally stable or unstable behavior (population oscillations) were double checked by applying an analysis of local stability, including linearization of the system (1)–(7) at the coexistence equilibrium and determining the stability properties by inspecting the corresponding eigenvalues (not shown).

## 3 RESULTS

In the absence of the plague, the crayfish population dynamics are locally stable. For the given rate of cannibalism, *c* = 1 × 10^−5^ the equilibrium susceptible adult population size settles down to the level *S* = 4320, and the ratio of the juveniles to the adults is *J*/*S* = 0.41.

### 3.1 Effects of environmental transmission compared to other transmission routes

**FIGURE 3:**
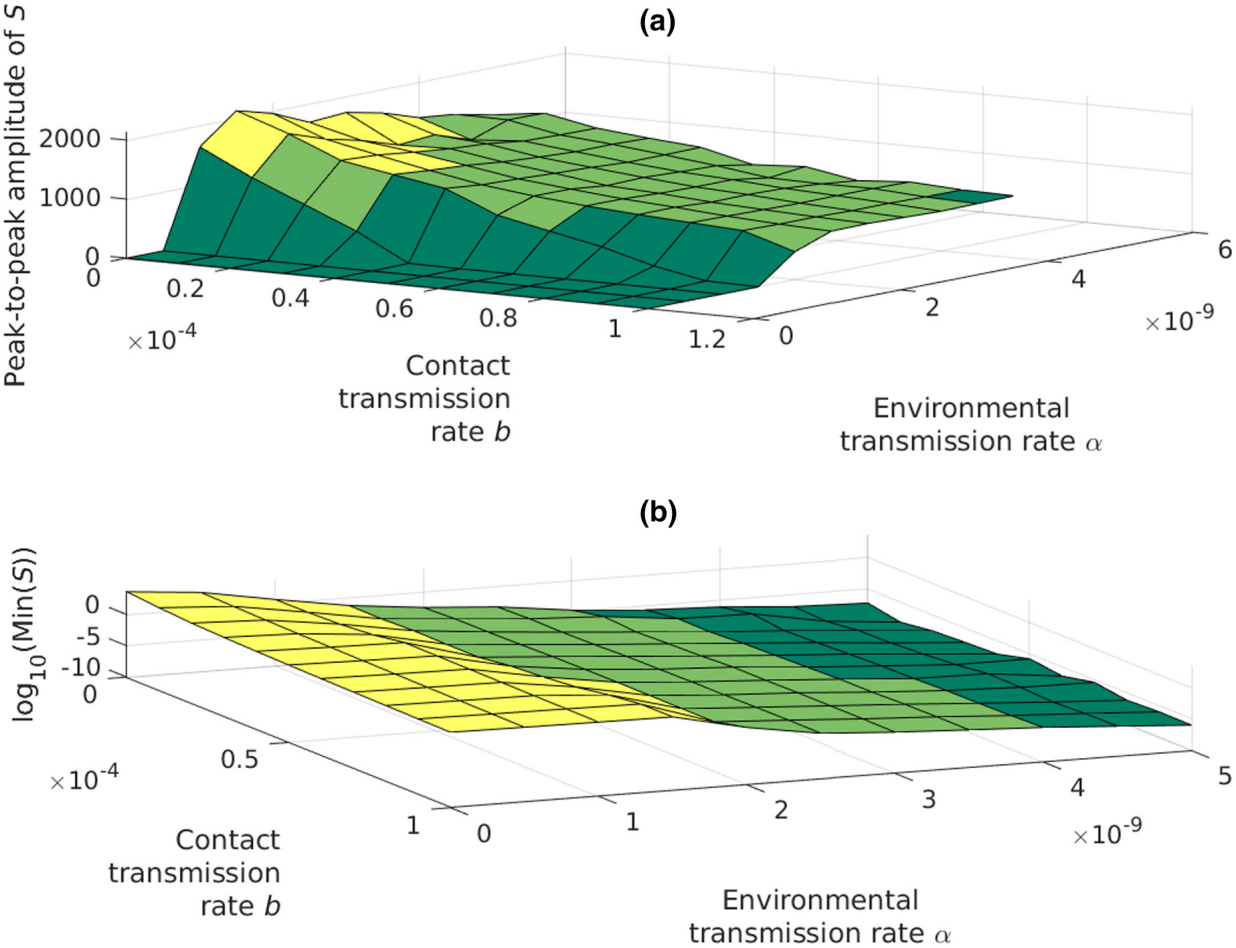
(a) the coexistence dynamics of the crayfish and the disease remain stable when the environmental transmission rate α is low. When the environmental transmission rate increases the coexistence dynamics turn abruptly into notable periodic oscillations. *Y*‐axis shows the amplitude of the periodic oscillations of the susceptible crayfish population. *Y*‐value of 0 means either that the crayfish population reaches a stable state, or the population goes extinct. (b) the minimum values of the oscillations decrease close to extinction with increasing the environmental transmission rate *α*. *Y*‐axis is log10 scaled to present a better view of the uneven gradient.

**FIGURE 4:**
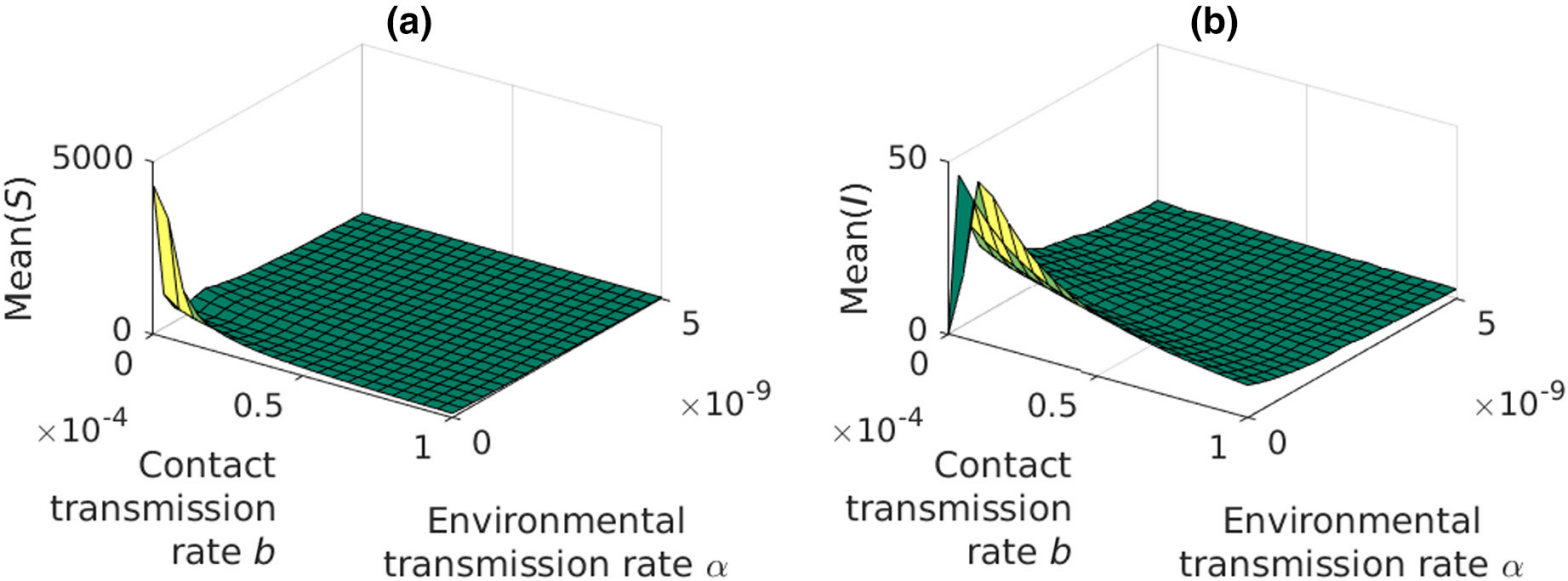
The effect of the environmental and contact transmission rates on the susceptible and infected adult crayfish populations. (a) the mean susceptible population size remains low if *Aphanomyces astaci* spreads through the environment, i.e., water. (b) while the environmental transmission rate remains low, an increase in the contact transmission rate decreases both the susceptible and the infected crayfish populations. To the contrary, at higher environmental transmission rate the mean infected crayfish population size remains greater with higher contact transmission rate. However, the overall population size of the crayfish decreases fast with increasing environmental transmission rate, thus shadowing the weak counter effect by the contact transmissions.

The coexistence dynamics between the crayfish and plague are locally stable when the environmental transmission rate *α* is low (Figure 2a), as is indicated by the area where peak‐to‐peak amplitude is zero. An increase in the environmental transmission rate turns the coexistence dynamics into heavy periodic oscillations (Figure 2a). At the same time, the minimum values of the crayfish population fluctuations decrease close to a local extinction (Figure 2b). Thus, a high environmental transmission rate prevents stable coexistence of the crayfish and the oomycete, but cyclic coexistence with population fluctuations is common. The contact infections strengthen the epidemic notably only if the environmental infection rate, *α*, is low (Figures 3a,b and 4).

**FIGURE 5:**
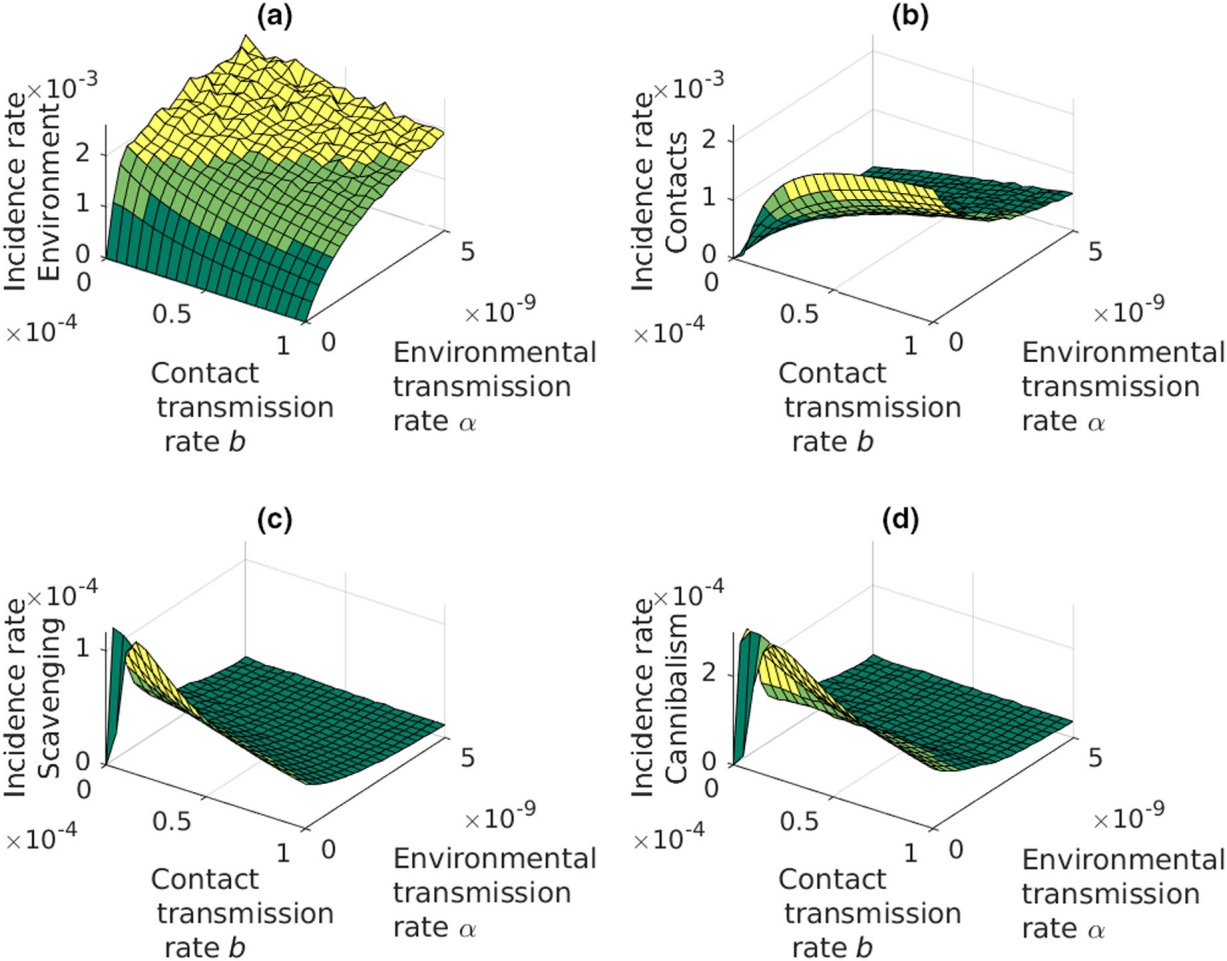
Strengths of the transmission routes. (a) Transmission through water is the strongest transmission route. Spikes in the figure are caused by surrounding local extinctions that prevent the propagation of *Aphanomyces astaci*. (b) Contacts remain the second most important route even at very low contact transmission rates. (c, d) scavenging and cannibalism remain very weak routes even when environmental and contact transmission rates are low. *Y*‐axis presents the incidence rate of new infected adult crayfish.

The infections from the environment have a major role in increasing disease epidemics (Figure 5). Environmental transmission dominates as an infection route except for very low values of the environment infection rate, *α* (Figure 5a). The other infection routes become meaningful only in the environment where the spores are rare. In this case, all the other infection routes remain low unless the rate of contact infections, *b*, increases. Low contact transmission rate together with high rate of environmental transmission causes slow periodic oscillations (Figure 5a). Even then the scavenging and cannibalism remain less important infection routes as compared to the contact infections. Cannibalism plays a minor role in spreading the infections (Figure 5d).

### 3.2 Effects of scavenging without environmental transmission

**FIGURE 6:**
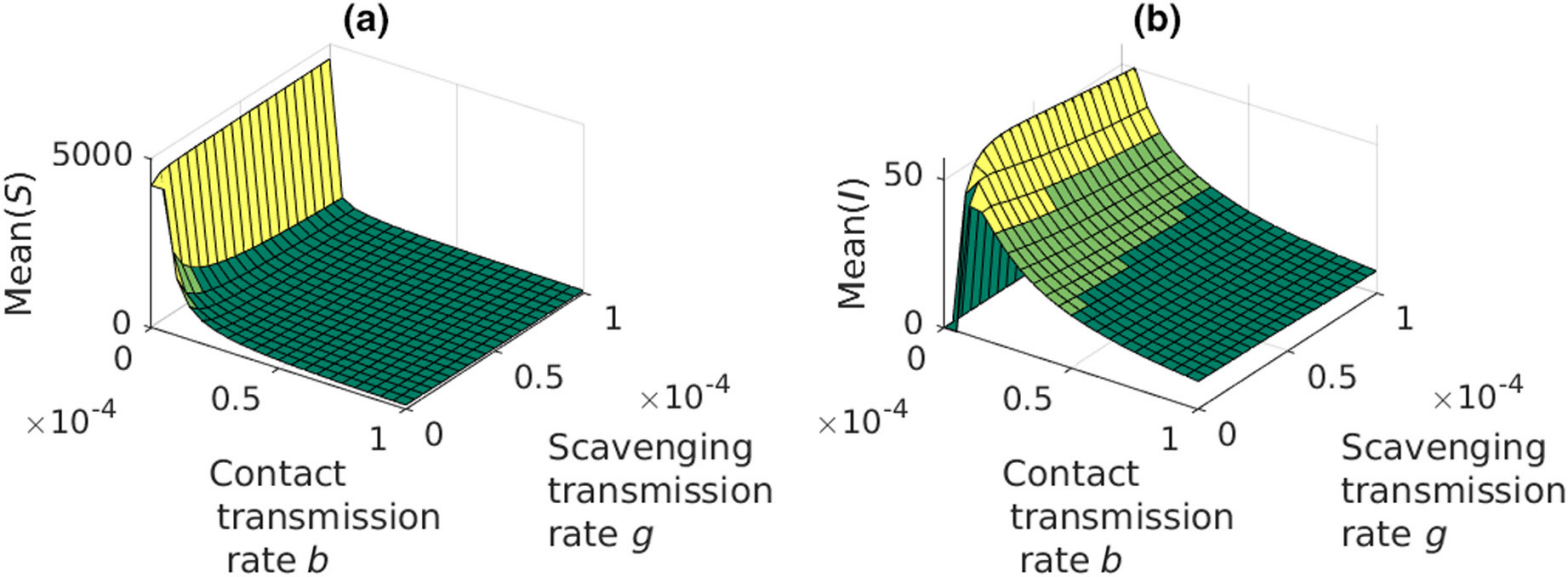
The mean population sizes of the susceptible and infected adult crayfish when the environmental infection rate is low (*α* = 1 × 10^−12^). Increasing contact transmission rate decreases the susceptible (a) and infected (b) crayfish population mean sizes. Scavenging has notable effect only when contact transmission rate is low, but higher than zero.

When we consider the effects of increasing rates of contact transmission and scavenging but keeping the environmental transmission almost absent (*α* = 1 × 10^−12^), we observe a disease‐free susceptible population at low contact transmission rate (*b* = 0; Figure 6a,b). When the contact transmission rate increases the size of the susceptible part of the population decreases quickly (Figure 6a). The size of the infected population first increases (Figure 6b), but then decreases when the contact transmission rate increases more.

**FIGURE 7:**
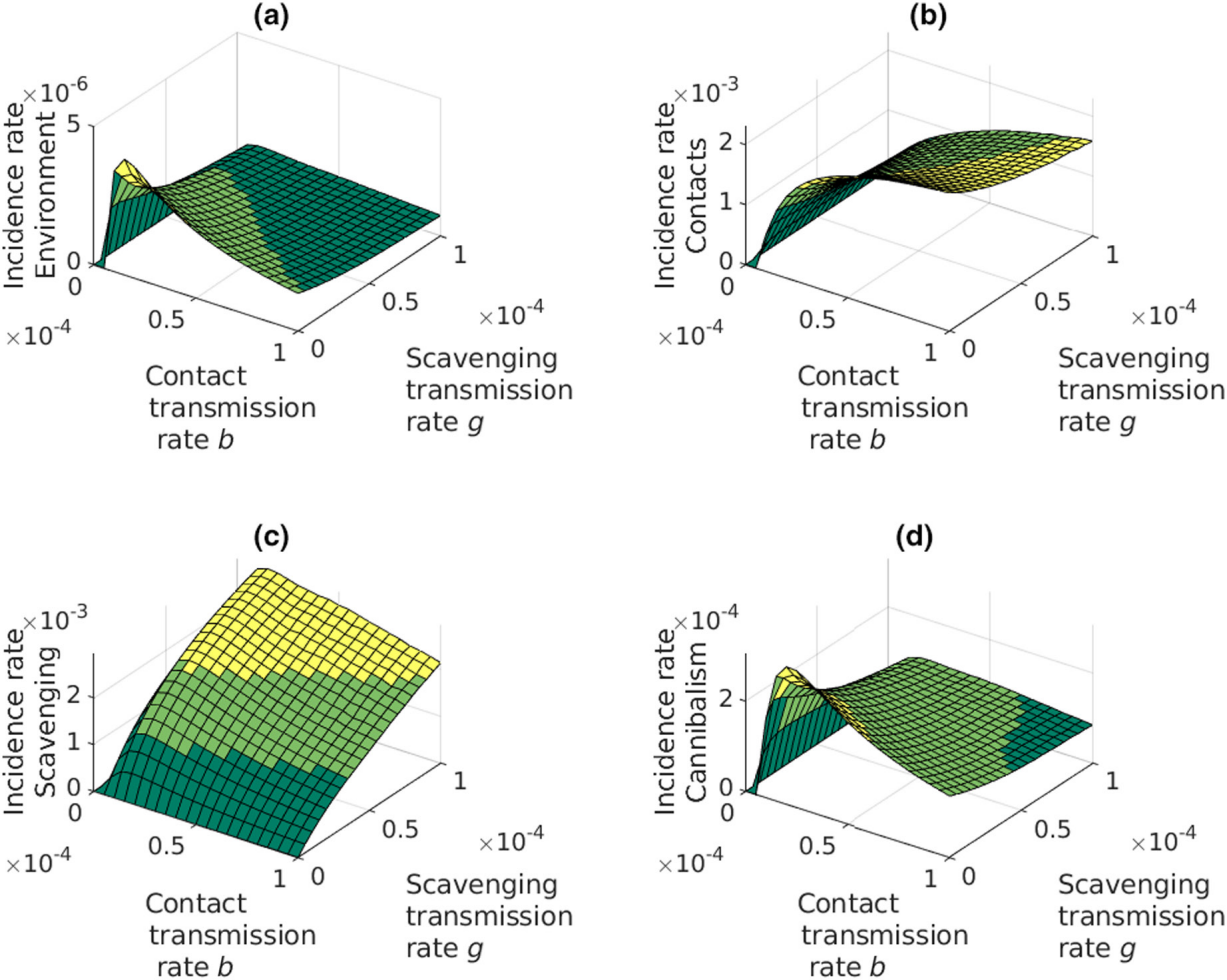
The incidence rates of the different infection routes of the plague when the environmental infection rate is low. (a) Environmental infection; (b) infections through contacts; (c) infections through scavenging; (d) infections through cannibalism. Incidence rate through scavenging increases with increasing scavenging rate. The effect of scavenging rate on the other incidence rates are most visible when contact transmission rate remains low, but above zero.

The environmental transmission route loses its strength due the low infection rate (Figure 7a), and the scavenging takes over the role as the leading source of infections when the rate of scavenging is higher than the rate of contacts (Figure 7c). Contact transmission route remains as a marked source of infections (Figure 7b), whereas cannibalistic infections are almost absent (Figure 7d). Increasing the rate of scavenging increases the incidence of infections but less than the increase in contact transmission rate.

### 3.3 Effects of cannibalism

**FIGURE 8:**
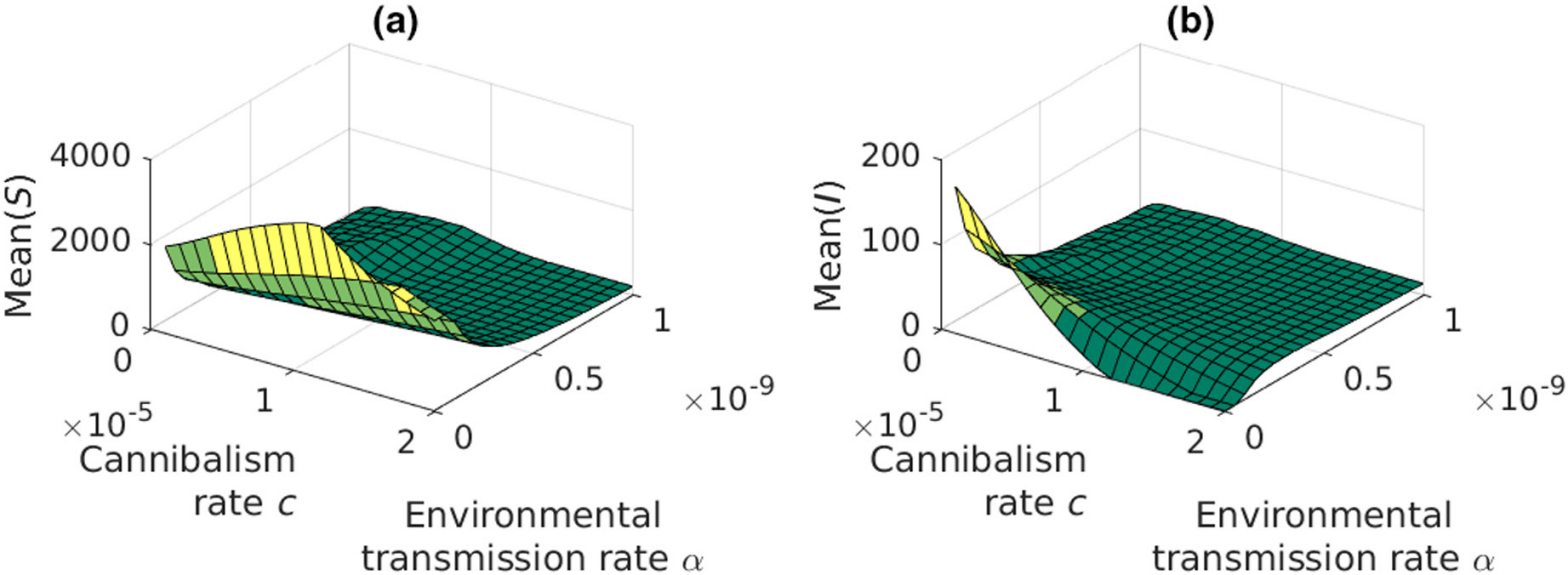
Mean population sizes of the (a) susceptible and (b) infected adult crayfish as a function of the environmental infection rate, *α*, and the infection rate through cannibalism, *c*. *α* and *c* have strong interaction, *c* acting as a stabilizing force. In the absence of environmental transmission, high rate of cannibalism can eradicate the pathogen from the system. However, even weak environmental transmission prevents the pathogens extinction.

An inspection of the role of cannibalism is challenging due to its dual role in the dynamics of infected crayfish populations. First, in the absence of the plague, cannibalism works as a regulating factor limiting the population size. Second, when the plague is present, cannibalism becomes an additional transmission route. The dual role of cannibalism becomes the clearest when the environmental infections are absent (Figure 8). When the pathogen is present, but the transmission rate through cannibalism is small (*c* = 1 × 10^−6^) the regulation by cannibalism and the infections through cannibalism, contacts, and scavenging maintain the susceptible population at low size (*S* = 2055, 4.6% of the pathogen‐free population size *S*_0_ = 44,844). When the rate of cannibalism increases, the mean size of the susceptible crayfish population increases to 3519, reaching 85.6% of the pathogen‐free population size with the same rate of cannibalism. At the same time, the number of infected crayfish decreases until complete vanishment. When the infected crayfish are gone, the regulation mechanism of the cannibalism begins to operate normally reducing the susceptible population size to *S* = 2138, which is equal to crayfish population size without the pathogen. In practice, the oomycete is completely eradicated. Thus, cannibalism regulates both the susceptible population and the prevalence of the disease.

If both contact transmission rate *b* and environmental transmission rate *α* are zero, no infected crayfish remain even with a very low cannibalism rate. Therefore, the susceptible population grows to the pathogen‐free population size. Without contact transmission, at low environmental transmission rate, the number of susceptible crayfish remains 20%–30% higher and the number of infected crayfish similarly lower than with added contact transmission. However, increasing the environmental transmission rate levels the difference, and already at *α* = 1 × 10^−9^ the difference is less than 10%.

When the environmental infection rate, *α*, increases it soon takes over the regulation of the population driving the mean size to a very low level (Figure 8a). If cannibalism rate is low, the dynamics are cyclic. Increasing cannibalism rate stabilizes the dynamics, higher *α* requiring higher cannibalism rate for the effect.

**FIGURE 9:**
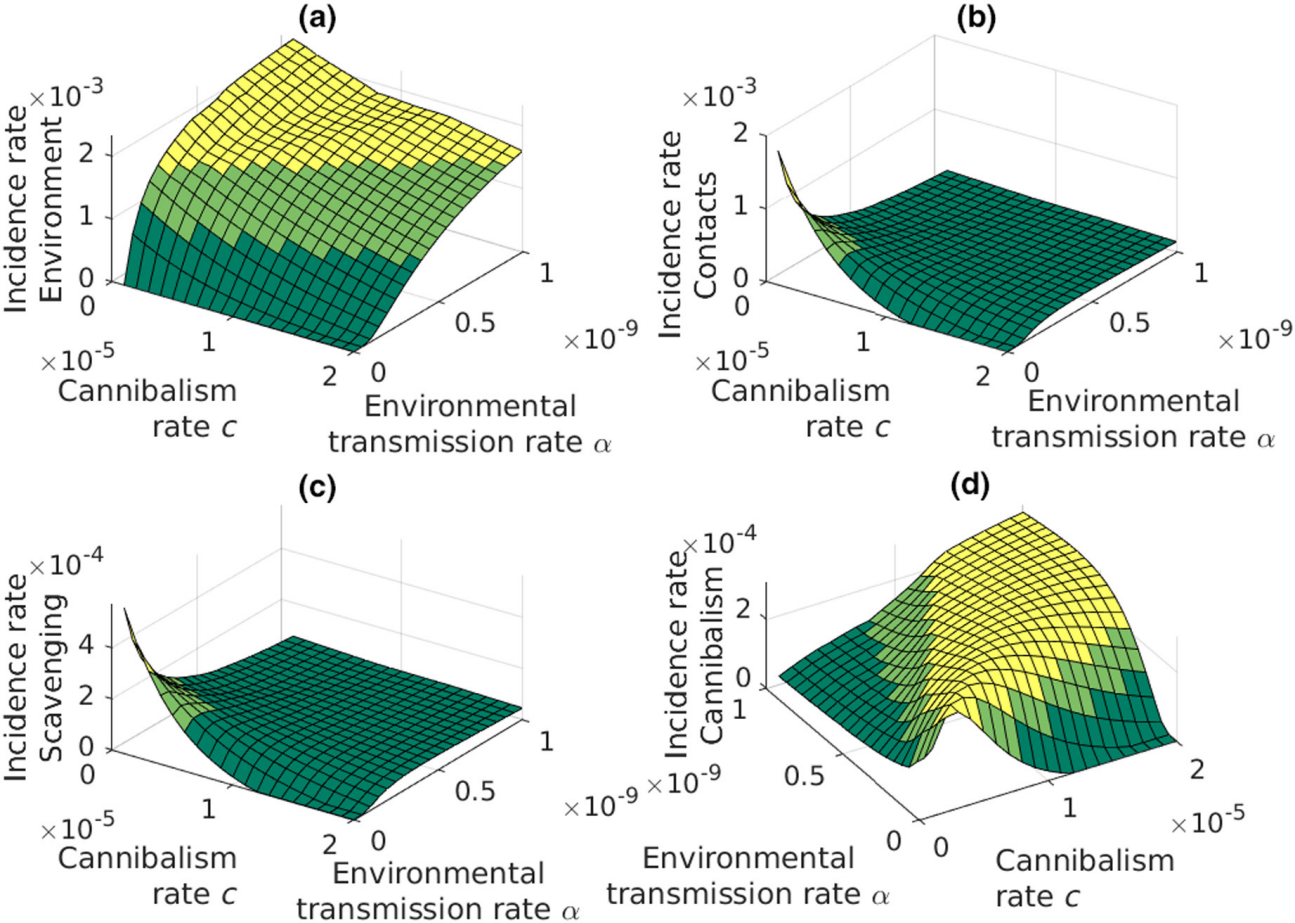
Incidence rates of the infections due to (a) environment, (b) contacts, (c) scavenging, and (d) cannibalism. Notice the different view angle in subfigure (d). In subfigure (a), one can see the stabilizing effect of increasing the rate of cannibalism, where at high environmental transmission rate the mean incidence rate is unstable at the area of low cannibalism rate. Subfigure (d) shows the strong interaction between the environmental transmission rate and the rate of cannibalism.

When the environmental transmission rate is zero, the respective incidence rate is obviously zero as well (Figure 9a). Increasing environmental transmission rate increases, however, the corresponding incidence rate until a possible extinction. Low rate of cannibalism together with high rate of environmental transmission causes slow periodic oscillations (Figure 9a). The incidence rates due to the contact infections (Figure 9b) and transmission through scavenging (Figure 9c) reach the highest peak when the environmental infection rate is zero and the cannibalistic infection rate is minimum (*c* = 1 × 10^−6^). Both decrease quickly with either increasing environmental infection rate or increasing cannibalistic infection rate.

## 4 DISCUSSION

Environmental transmission including transmission through water and surfaces forms a natural route for the pathogen (Oidtmann et al., 2002). According to our model, environmental transmission is the decisive mechanism in the development of disease epidemics. Compared with a pathogen‐free population, a low environmental transmission rate decreases the crayfish population size, but only with a low risk of extinction, regardless of the contact transmission rate. Higher environmental transmission rate, however, substantially increases the number of infected crayfish, potentially driving both the crayfish and pathogen populations to extinction. On the other hand, high contact transmission rate creates probability for cyclic population fluctuations with low mean population size, though the dynamics are strongly dependent on the initial size of the infected population. As a result, a high contact transmission rate with a low but nonzero environmental transmission rate can lead to extinction, cyclic population with a minimum close to extinction, or large susceptible crayfish population. In accordance with our model, *A. astaci* has been reported to survive even years after an outbreak within a low‐density crayfish population (Viljamaa‐Dirks et al., 2011). In natural conditions, the relative importance of environmental and contact infections could depend on the habitat complexity so that contact infections might dominate in environments where the crayfish distribution is very patchy. Thus, they may not be biologically irrelevant but induce dynamics that do not obey standard density‐dependent theory as suggested by our model at low environmental transmission rates.

Environment remained the most obvious transmission route for *A. astaci* in most scenarios included in our simulations. However, Cerenius and Söderhäll (1984) reported that in turbulent water, *A. astaci* spores formed cysts that are inactive but long lived and can transform back to infectious spores. Therefore, high water turnover, as in fast flowing streams, could decrease the importance of environmental transmission. Similarly, in initial stage of an outbreak, the spore density in water is low (Strand et al., 2014). Further, Svoboda et al. (2017) report in their review cases of potential contact transmissions. To this end, in some cases, contact transmission, and especially transmission through necrophagy, could gain more significance compared to the environmental transmission. In our simulations, increasing the contact transmission rate at high environmental transmission rate increases the number of infected crayfish. However, the effect is weak compared to the overall population decline caused by the increasing environmental transmission rate. In practice, low environmental transmission rate in our model corresponds to a pathogen spore density that stays below the possible infection threshold in a natural system (Makkonen et al., 2014). Thus, the contact transmission could be even more significant in nature than in our model that excludes the potential non‐linearity in environmental transmission rate. Our model reveals the potential significance of the different frequency‐dependent transmission routes, and further field data on contacts between healthy and infected crayfish are needed to test the plausibility of the theoretical predictions.

Scavenging has a marked role only if the environmental transmission rate is low. Specifically, scavenging the dead conspecifics does not have a clear effect on the population size even with the relatively long decay rates for the infective carcasses used in the simulations. This is plausible because *A. astaci* produces motile zoospores at the fastest rate during the time right before and a few days after the death of the host. The competition for the carcasses between scavenger species can be strong, while the decomposition process is relatively fast in submerged carcasses (Anderson et al., 2019; Beasley et al., 2012; Fenoglio et al., 2014). Therefore, the effective scavenging time for the crayfish carcasses is limited, which affects the efficiency of carcasses as infection sources. However, empirical data on the process are scarce and urgently needed. Consequently, the significance and the action of intraspecific necrophagy as a part of population dynamics are currently not thoroughly understood.

In the present model, cannibalism stabilizes the host population dynamics by reducing the amplitude of the population cycles. Cannibalism can also slow down the epidemic by two mechanisms. First, the attacker removes the infection transmitter from the system, thus decreasing the contact infections. Second, by removing the diseased individuals before they reach the state where *A. astaci* spore production is at its highest, cannibalism reduces the effect of the environmental transmission. Therefore, the effect of cannibalism on the population is positive, even though it repeats the cycle of transmission. Importantly, increasing cannibalism can eradicate the oomycete completely if the environmental transmission rate is very low. This suggests a role for evolutionary group selection such that cannibalistic groups could be more efficient in resisting diseases than non‐cannibalistic groups. However, the feasibility of this hypothesis would require further analysis and testing.

Abrahamsson (1966) suggested that a crayfish population can be regulated by cannibalism of large males. This hypothesis was supported by Houghton et al. (2017), who verified in *P. leniusculus* that cannibalizing individuals were primarily notably larger than the victims. To our knowledge, the population dynamical consequences of this have not been studied theoretically. Polis (1981) names intraspecific predation as a homeostatic factor possibly influencing population structure, life history, competition for mates and resources, and other behavior, among many animals. In his review (see also Fox, 1975), he mentions that cannibalism occurs in (at least in) about 1300 species. In our simple model, cannibalism limits the population growth such that population size declines with increasing intensity of intraspecific predation and population size is always stable over time. No other density‐dependent self‐regulating factors are needed to maintain stable population size (Rudolf & Antonovics, 2007). In size‐structured models with density‐dependent recruitment, also other types of solutions (cyclic dynamics) are possible. Because our model did not have carrying capacity or density‐dependent recruitment, the modeled two contradictory processes necessarily lead to a stable equilibrium in a disease‐free system.

When *A. astaci* is introduced to the system, be it through non‐indigenous crayfish, digestive tract of fish, contaminated equipment, or infected pieces of crayfish carcasses, its transmission modes are versatile (Oidtmann et al., 2002; Svoboda et al., 2017). Therefore, the epidemiological model needs to extend the classical SI model with both environmental and vector borne transmissions (Anderson & May, 1981). The combination of transmission routes causes complexity where the dynamics of the epidemic vary considerably according to the model parameters. The different pathogen transmission modes have only partly overlapped effects on the host population size and the periodicity of the dynamics.

Although *A. astaci* displays diversity in virulence and host specificity, in all strains the zoospore dose is likely the most important factor in determining the probability and severity of infection (Makkonen et al., 2014). Our model assumed a non‐evolving pathogen strain. Together with the apparent inability of the crayfish to acquire immunity against the pathogen (c.f. Gruber et al., 2014), the model presented here predicts periodic population cycles with moderate environmental infection rates. Complete periodic cycles have not been reported, but virulent *A. astaci* has been reported to survive years after an outbreak within a very weak crayfish population, causing recurrent epidemics among restocked crayfish populations (Viljamaa‐Dirks et al., 2011). These recurrent epidemics might be caused by a very small number of surviving crayfish that were still carrying the oomycete, when the population was falsely regarded to be extinct before restocking. Even among the highly susceptible noble crayfish, there are likely populations that tolerate infections by certain *A. astaci* strains (Jussila et al., 2014, 2015). The resistance against the pathogen in North American crayfish can be attributed to coevolution between the crayfish and the endemic pathogen (Svoboda et al., 2017). Elevated mortality (Aydin et al., 2013; Thomas et al., 2020) and declined fecundity due to eroded swimmeret syndrome caused by chronic infection in some European signal crayfish populations (Jussila et al., 2017) support this assumption with reciprocal examples.

When environmental transmission rate is very low, there are interesting similarities between our model, the spread of crayfish plague in Europe, African swine fever (ASF) in Europe and Asia, and the Ebola virus disease (EVD) in Western Africa. ASF and EVD incorporate the potential environmental transmission through reservoir species, but the epidemics rely heavily on the transmission through the frequency‐based contacts with dead individuals. ASF has been modeled using purely density‐dependent contact infection model (Barongo et al., 2016), but the disease dynamics can be modeled with several transmission routes similarly to the crayfish plague (O'Neill et al., 2020). Transmission routes are similar to crayfish plague, including contact and environmental infections. Cannibalism has also been shown to be part of the ASF dynamics (Cukor et al., 2020), though not as a population regulator. In the EVD epidemic, the transmission through the dead was caused by the tradition of touching the deceased as a part of burial ceremonies. The tradition, which is present in many human cultures, was an important transmission route during the West African Ebola epidemic (Manguvo & Mafuvadze, 2015). To the contrary, in areas where EVD is endemic the infected bodies are avoided, reducing the transmission rate. Environmental transmission of ASF and EVD happen through reservoir animals and contaminated resources. To this end, these examples show that, despite some obvious differences in the scenarios, the multilayered transmission dynamics of crayfish plague captured by our model can be generalized to other diseases and populations.

The control of the crayfish plague is considered exceptionally challenging due to the strong effect of the crayfish behavior on the population dynamics and the most complicated disease dynamics of the crayfish plague. Thus, the debate about the disease control is almost absent in the scientific literature, mainly due to the lack of adequate theory to master the disease dynamics in crayfish populations. Although some empirical methods have been tested, they have not yielded clear successes (Jussila et al., 2021). Toward this end, we have presented here a new population dynamical model that includes the peculiarities of the crayfish life history and behavior, specifically cannibalism and necrophagy. We combined the crayfish population model with the disease dynamics with four transmission routes: waterborne transmission, contact transmissions, transmission through cannibalism, and transmission through necrophagy as part of scavenging. This allowed us to analyze the effect of the different disease infection routes to the quality of the dynamics of an infected crayfish population. Our model illustrates that mean crayfish population sizes depend on type of the pathogen transmission. The present model can potentially be further modified and applied in the conservation and management of crayfish populations. The details of the crayfish and disease dynamics are expected to vary considerably due to the locally varying environmental conditions and the biogeography of the different crayfish species.

## AUTHOR CONTRIBUTIONS

**Mikko Koivu‐Jolma:** Conceptualization (equal); investigation (equal); visualization (supporting); writing – original draft (equal); writing – review and editing (equal). **Raine Kortet:** Conceptualization (equal); writing – review and editing (equal). **Anssi Vainikka:** Conceptualization (equal); writing – review and editing (equal). **Veijo Kaitala:** Conceptualization (equal); formal analysis (lead); investigation (equal); supervision (lead); visualization (lead); writing – original draft (equal); writing – review and editing (equal).

## CONFLICT OF INTEREST

We declare no conflict of interest.

## Data Availability

Data sharing not applicable to this article as no datasets were generated or analysed during the current study.
